# Retinal microvascular abnormalities overlying choroidal nodules in neurofibromatosis type 1

**DOI:** 10.1186/1471-2415-14-146

**Published:** 2014-11-25

**Authors:** Solmaz Abdolrahimzadeh, Lorenzo Felli, Domenica Carmen Piraino, Roberto Mollo, Stefano Calvieri, Santi Maria Recupero

**Affiliations:** Ophthalmology Unit, DAI Testa/Collo, Azienda Policlinico Umberto I, University of Rome “Sapienza”, viale del Policlinico 155, Rome, Italy; Ophthalmology Unit, Organi di Senso Department, University of Rome “Sapienza”, viale del Policlinico 155, Rome, Italy; Dermatology Unit, University of Rome “Sapienza”, viale del Policlinico 155, Rome, Italy; Ophthalmology Unit, Sant’Andrea Hospital, NESMOS Department, University of Rome “Sapienza”, Via di Grottarossa 1035/1039, Rome, Italy

**Keywords:** Neurofibromatosis type 1, Retinal microvascular alterations, Near infrared reflectance, Optical coherence tomography-enhanced depth imaging, Phakomatoses

## Abstract

**Background:**

Neurofibromatosis type 1 (NF1) is an autosomal dominant disorder involving aberrant proliferation of multiple tissues of neural crest origin. Retinal vascular alterations in NF1 have rarely been reported in the literature and their nature is not clear. This study describes distinctive retinal microvascular alterations and their relationship to choroidal nodules in patients with neurofibromatosis type 1.

**Methods:**

This was a retrospective study where records of seventeen consecutive patients with diagnosis of NF1, presenting Lisch nodules and choroidal alterations, and 17 age and gender-matched healthy control patients were evaluated. Fundus photographs, near infrared reflectance and enhanced depth imaging - optical coherence tomography images were reviewed. Retinal microvascular abnormalities and choroidal and retinal alterations in proximity of the retinal microvacular alterations were carefully noted.

**Results:**

6 patients (35%) presented distinctive microvascular abnormalities. These consisted of small, tortuous vessels with a “spiral” or “corckscrew” aspect. They were second or third order, small tributaries of the superior or inferior temporal vein. These vessels were all located overlying choroidal alterations as observed with near infrared reflectance. Enhanced depth imaging - optical coherence tomography showed alteration of choroidal vasculature due to the presence of choroidal nodules but otherwise retinal and choroidal cross-sections were unremarkable for morphology.

**Conclusions:**

Retinal microvascular alterations overlying choroidal nodules in patients with NF1 can be considered another distinctive characteristic of the disease. Although the nature of these alterations is not clear, the authors speculate that functional disorders of vasomotor nerve cells, which originate in the embryonal neural crest can lead to their formation.

## Background

Neurofibromatosis type 1 (NF1) is part of a group of heredofamilial disorders characterized by the presence of disseminated hamartoma for which Van der Hoeve introduced the termed “phakomatoses” in 1923 [1]. In ophthalmology hamartoma of the iris, termed Lisch nodules, are among the diagnostic criteria of NF1 [2]. However, choroidal hamartomas have also been proposed since they occur in 82 to 100% of cases [3, 4]. In a unique report by Muci-Mendoza et al. retinal microvascular abnormalities were observed in 37.5% of patients [5]. The nature of these vessels is not clear, although it has been hypothesized that, due to their stable nature, they may be congenital. The present study was carried out to evaluate the presence of retinal microvascular abnormalities in patients with NF1 and to determine if these were associated with choroidal alterations.

## Methods

All persons gave their informed consent prior to inclusion in the study. The research was given approval by the Institutional Review Board of the University of Rome “Sapienza” and was conducted in accordance with the tenets of the Declaration of Helsinki. Records of seventeen consecutive patients examined in 2014 with diagnosis of NF1 based on the stringent National Institutes of Health diagnostic criteria [6] were evaluated. Records of 17 age and gender-matched healthy control patients were also examined. Fundus photographs, near infrared reflectance (NIR) and enhanced depth imaging (EDI) optical coherence tomography (OCT) images acquired with the Spectralis OCT Family Acquisition Module, V 5.1.6.0 Heidelberg Engineering, Germany were reviewed. Retinal microvascular alterations and choroidal or retinal alterations in proximity of the microvascular alterations were carefully noted.

## Results

**Figure 1:**
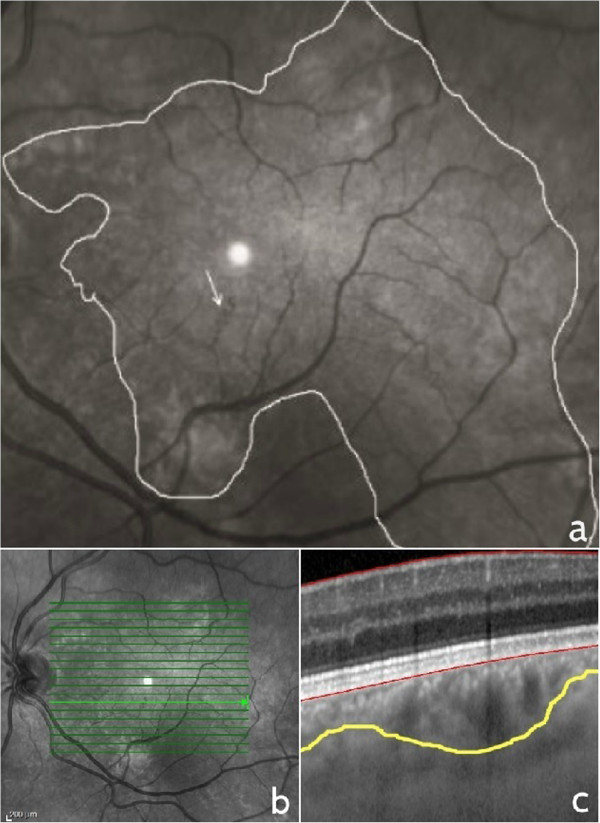
**Near infrared reflectance and enhanced depth imaging optical coherence tomography cross-section of retinal and choroidal alterations in NF1.** Near infrared reflectance image of “corkscrew” or “spiral” microvascular alteration (arrow) overlying large patchy choroidal alteration delineated with a white line **(a)**, raster optical coherence tomography image and corresponding cross-section of the choroidal nodule delineated with a yellow line **(b and c)**.

**Figure 2:**
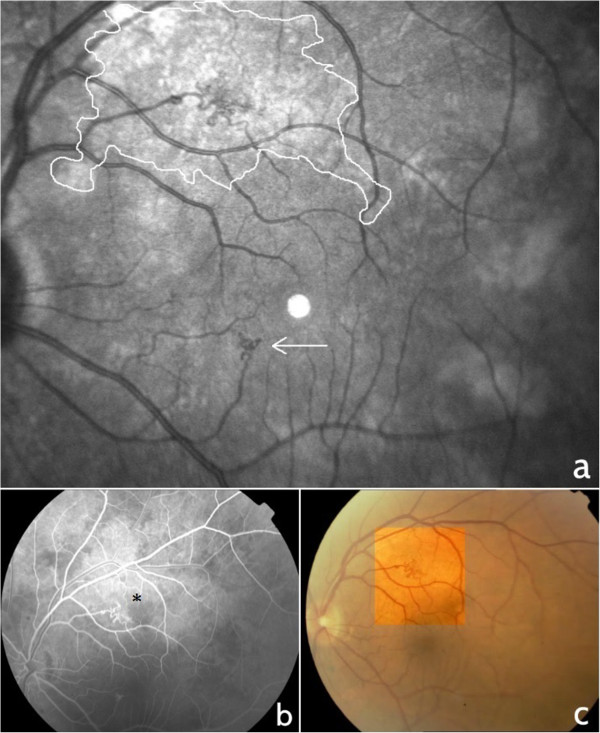
**Retinal microvascular abnormality with “hemangioma-like” or “ball of thread” appearance in NF1.** Near infrared reflectance image where the white line delineates the patchy choroidal alteration around the hemangioma-like microvascular abnormality and the arrow indicates a smaller microvascular abnormality **(a)**, fluorescein angiography where the asterisk indicates the hemangioma-like microvascular abnormality **(b)** and fundus photograph with colour enhancement of the microvascular alteration **(c)**.

Microvascular retinal abnormalities were identified in 6 patients (35%) (3 men and 3 women, 42 to 57 years of age) in a total of 17 patients with NF1 presenting Lisch and choroidal nodules. No similar alterations were identified in the healthy control patients. The microvascular alterations were localized in the vascular arcade area and were second or third order, small tributaries of the superior or inferior temporal vein. They consisted of small tortuous vessels with a “spiral” or “corkscrew” appearance in 3 female and 2 male patients (Figure 1). One male patient, with extensive cutaneous neurofibromata, had 2 microvascular alterations of the superior and inferior temporal vein with a more complex “hemangioma-like” or “ball of thread” aspect (Figure 2). Fluorescein angiography images in this case showed that the microvascular alteration did not leak fluorescein. Visual acuity was 20/20 in all patients.

NIR images showed that the retinal microvessels were all overlying patchy choroidal alterations. On cross-sectional EDI-OCT images these alterations corresponded to hyperreflective choroidal nodules which occupied space in the choroidal thickness and gave an irregular profile to the choroidal vasculature.

## Discussion

Retinal angiomatosis in a patient with NF1 was reported in 1967 by Frenkel [7]. Muci-Mendoza et al., in 2002, reported on 12 of 32 patients (37.5%) who presented retinal microvascular abnormalities [5]. The present report is the second case series showing retinal microvascular alterations in six of 17 NF1 patients (35%).

In the present study, all abnormal retinal microvessels were overlying patchy choroidal alterations as shown on NIR imaging. However, they were localized to the retina and did not involve the choroid, as shown by spectralis EDI-OCT cross-sections. Although choroidal vasculature is altered by the space occupying choroidal nodules, it is difficult to establish whether there is any correlation between these nodules and the abnormal microvascular retinal vessels.

Choroidal nodules are ovoidal bodies consisting of proliferating Schwann cells arranged in concentric rings around an axon [8]. They show similarities to cutaneous neurofibromata and Lisch nodules of the iris [9]. This follows the principle of aberrant proliferation of tissues of neural crest origin in NF1 [10] but does not directly justify the presence of vascular abnormalities, which can be an expression of changes in mesodermal germ layers**.** The term “phakomatoses”, as described by Van der Hoeve, includes Recklinghausen’s neurofibromatosis, tuberous sclerosis, Hippel-Lindau disease, Sturge-Weber syndrome, Louis-Bar syndrome and Wyburn-Mason syndrome [1]. Even before the modern era of DNA genomic studies, similarities in inheritance patterns and affected germ layers had been noted among the various phakomatoses. Today, there is still wide discussion regarding the interrelationships between these forms. Sporadic reports in the literature show that a relationship can exist between a chiefly ectodermal and a primarily mesodermal phakomatosis and NF1 has been reported in association with von Hippel’s disease [11, 12]. Furthermore, in phakomatosis pigmentovascularis there is an association of vascular and pigmentary alterations [13, 14]. There is evidence supporting developmental abnormalities of vasomotor nerve cells that originate in the embryonal neural crest and immunohistochemical studies have shown the presence of perivascular nerves in the port wine stains seen in Sturge-Weber Syndrome [15]. Although this is an extremely complex field where both embryogenesis and genomic analysis must be taken into consideration, it can be speculated that functional disorders of vasomotor nerve cells which originate in the embryonal neural crest can lead to the retinal microvascular alterations observed in NF1 patients.

## Conclusions

This study describes “spiral” or “corkscrew” retinal microvascular alterations overlying choroidal nodules in patients with NF1. These alterations can be considered a distinctive retinal feature of NF1. Furthermore, as NF1 involves aberrant proliferation of multiple tissues of neural crest origin, these retinal alterations could possibly be related to functional disorders of vasomotor nerve cells.

## Abbreviations

NF1: Neurofibromatosis type 1
NIR: Near infrared reflectance
OCT: Optical coherence tomography
EDI: Enhanced depth imaging.
